# Energy identity and no neck property for $\varepsilon$-harmonic and $\alpha$-harmonic maps into homogeneous target manifolds

**DOI:** 10.1007/s00526-025-03233-w

**Published:** 2026-01-12

**Authors:** Carolin Bayer, Andrew M. Roberts

**Affiliations:** 1 Department of Mathematics, ETH Zentrum, CH-8093 Zurich, Switzerland; 2 School of Mathematics, University of Leeds, Leeds, LS2 9JT United Kingdom

## Abstract

In this paper we show the energy identity and the no-neck property for $\varepsilon$- and $\alpha$-harmonic maps with homogeneous target manifolds. To prove this in the $\varepsilon$-harmonic case we introduce the idea of using an equivariant embedding of the homogeneous target manifold.

## Introduction

Let $(M^2,g)$ be a smooth, compact Riemannian surface without boundary and $(N^n,h)$ be a compact Riemannian manifold without boundary which we assume to be equipped with an isometric embedding into Euclidean space $N\hookrightarrow \mathbb {R}^l$. Define for $u\in W^{1,2}(M,N)$ the Dirichlet energy

$$\begin{aligned} E_0[u]=\frac{1}{2}\int _{M}\vert \nabla u\vert ^2. \end{aligned}$$

We call *u* harmonic if it is a critical point of the energy functional and these maps satisfy

$$\begin{aligned} \Delta u = A(u)(\nabla u,\nabla u), \end{aligned}$$

where $A(u)(X,Y)=(\nabla _XY)^\perp \in (T_uN)^\perp$ is the second fundamental form of the embedding $N \hookrightarrow \mathbb {R}^l$. The Dirichlet energy does not obey the Palais-Smale condition, so various approximations to the energy functional have been introduced which allow us to use methods from the calculus of variations. In [19] Sacks and Uhlenbeck introduced the notion of $\alpha$-energy defined for $\alpha >1$ by

1 $$\begin{aligned} \bar{E}_\alpha [u]=\frac{1}{2}\int _{M} \big ((1+|\nabla u|^2)^\alpha -1\big ) \end{aligned}$$

and in [8] Lamm introduced the notion of $\varepsilon$-energy defined for any $\varepsilon >0$ by

2 $$\begin{aligned} \tilde{E}_\varepsilon [u]= \frac{1}{2} \int _M \big ( |\nabla u|^2+\varepsilon |\Delta u|^2 \big ). \end{aligned}$$

Both these functionals obey the Palais-Smale condition so critical points exist, and can be shown to be smooth. We call these critical points $\alpha$-harmonic (resp. $\varepsilon$-harmonic). Note that the Euler-Lagrange equation of (1) can be written as

3 $$\begin{aligned} {{\,\textrm{div}\,}}(F_\alpha \nabla u_\alpha )&= F_\alpha A(u_\alpha )(\nabla u_\alpha , \nabla u_\alpha ) , \end{aligned}$$

where $F_\alpha := (1+\vert \nabla u_\alpha \vert ^2)^{\alpha -1}$. The Euler-Lagrange equation of (2) is given by

$$\begin{aligned} \Delta u - \varepsilon \Delta ^2 u&= \sum \limits _{i=n+1}^l \varepsilon \big ( \Delta (\langle \nabla u, \nabla \nu _i|_u(\nabla u) \rangle ) + {{\,\textrm{div}\,}}(\langle \Delta u, \nabla \nu _i|_u(\nabla u) \rangle ) \nonumber \\&\quad + \langle \nabla \Delta u , \nabla \nu _i|_u(\nabla u) \nabla u \rangle \big ) \nu _i (u) - A(u)(\nabla u, \nabla u), \end{aligned}$$

where $\{ \nu _i \}_{i=n+1}^l$ is a smooth local orthonormal frame for the normal space of $N \subset \mathbb {R}^l$ near *u*(*x*). Note that this is equivalent to

4 $$\begin{aligned} (\Delta u - \varepsilon \Delta ^2 u)^\top =0 . \end{aligned}$$

If we now take a sequence $u_k$ of $\alpha _k$-harmonic (resp. $\varepsilon _k$-harmonic) maps with $\alpha _k\rightarrow 1$ (resp. $\varepsilon _k\rightarrow 0$) with uniformly bounded $\alpha _k$-energy (resp. $\varepsilon _k$-energy) then in both cases it is clear that there exists some weakly harmonic map $u_0$ such that $u_k\rightharpoonup u_0$ weakly in $W^{1,2}(M,N)$. Further, $u_0$ will be smooth with $C^m_{\text {loc}}$ convergence for any *m* away from a finite set $\Sigma$ and at the points in $\Sigma$, in both cases, the maps will undergo the standard bubbling procedure.

The two main questions that arise from the bubbling procedure are those of potential energy loss and neck formation. We would like to show that there is no energy loss or oscillation in the neck region. There are many previous results related to this type of energy approximation, some of which we will now discuss. In [18] Parker showed that sequences of harmonic maps into compact Riemannian manifolds also obey the same bubbling procedure and have both the no energy loss and no neck properties, in [8] Lamm showed that sequences of $\varepsilon$-harmonic maps into round spheres have the no energy loss property and in [13] Li and Zhu showed that sequences of $\alpha$-harmonic maps into round spheres have the no energy loss and the no neck properties. Their result is fundamentally based on the duality of $(L^{2,1})^* = L^{2,\infty }$

$$\begin{aligned} \Bigg \vert \int \limits _U f \cdot g \; \Bigg \vert \le \Vert f\Vert _{L^{2,1}(U)} \cdot \Vert g \Vert _{L^{2,\infty }(U)}. \end{aligned}$$

The importance of this duality and its applications to so-called neck analysis was first stressed and exploited by Lin and Rivière in [16] where they derive an energy-defect identity for sequences of stationary harmonic maps from higher dimensional domains into spheres. This result was systematised by Rivière and Laurain in [11] for elliptic linear systems with antisymmetric potentials in two dimensions.

Regarding the regularised energy $\bar{E}_\alpha$, Chen and Tian gave in [1] the energy identity for minimising sequences which map into general target manifolds for a given homotopy class. Furthermore, Li and Wang proved in [14] a generalised energy identity for $\alpha$-harmonic maps again into general target manifolds and studied the length of necks. In particular, in the case where there is only one bubble, they gave an explicit length formula for the neck. In addition, Lamm [9] proved under certain entropy-type condition that for $\alpha$- and $\varepsilon$- harmonic sequences no energy loss occurs. He studied Hopf-differential-type structures in the two approximations. Da Lio and Rivière reformulated in [3] for *p*-harmonic systems with anti-symmetric potentials their divergence form as a conservation law. They provided a sufficient condition for controlling the length of the necks at the limit to a much wider case of PDEè?s including p-harmonic relaxation of arbitrary conformally invariant Lagrangians.

Jost, Liu and Zhu [6] consider a sequence of maps from a compact Riemann surface with smooth boundary which map into a general compact Riemannian manifold with free boundary on a smooth submanifold. In addition, this sequence has a uniformly bounded energy and tension field. They then showed that the energy identity and the no-neck property hold during a blow-up process. Under these assumptions, they considered the Sacks-Uhlenbeck $\alpha$-harmonic case in [7] and proved a generalised energy identity. Further, for $\alpha$-harmonic maps in the spherical target manifold case, Li, Zhu and Zhu [12] showed that the no-neck property additionally holds.

We will now show both the no energy loss and no neck property for sequences of $\alpha$-harmonic maps and of $\varepsilon$-harmonic maps in the case where the target manifold is a compact homogeneous manifold. A Riemannian manifold (*N*, *h*) is defined to be homogeneous if the action of its Lie group of isometries *G* is transitive. In [4] Hélein introduced the idea of using Killing vector fields induced by the Lie algebra of *G* to work with homogeneous manifolds. For any $a\in \mathfrak {g}$ we have a Killing vector field $\rho (a)\in \Gamma (TM)$ induced by *a*.

In the case of $\alpha$-harmonic maps, for any $\psi \in G$ we can show that $\bar{E}_\alpha [\psi \circ u]=\bar{E}_\alpha [u]$, due to the fact that $\bar{E}_\alpha$ is an intrinsic property. Then by Noether’s theorem we obtain a conservation law similar to the one derived in [13] for the sphere, following Hélein’s approach for harmonic maps. Then by modifying their arguments one can show the no energy loss and no neck property.

However, this does not work in the case of $\varepsilon$-harmonic maps, since $\tilde{E}_\varepsilon [\psi \circ u]$ generally does not equal $\tilde{E}_\varepsilon [u]$. The Laplacian depends on the embedding of *N*, so $|\Delta u|$ is generally not equal to $|\Delta ( \psi \circ u)|$. To account for this, we will use a specific type of embedding, the existence of which was shown in [17] by Moore.

### Theorem 1.1

Any homogeneous Riemannian manifold (*N*, *h*) admits an isometric and equivariant embedding into some Euclidean space.

In particular we have that if (*N*, *h*) is a homogeneous Riemannian manifold with *G* its Lie group of isometries, there exists $\Phi :N\rightarrow \mathbb {R}^l$ an isometric embedding and $\Pi :\text {Isom}(N)=G\rightarrow O(l)$ an embedding such that

$$\begin{aligned} (\Pi (\psi ))(\Phi (q))=\Phi (\psi (q)) \end{aligned}$$

for any $\psi \in G,q\in N$. In other words, any intrinsic isometry of *N* is induced by some extrinsic isometry of the entire Euclidean space. In this case, we easily get $\tilde{E}_\varepsilon [\Pi (\psi )\circ u]=\tilde{E}_\varepsilon [u]$. Then Noether’s theorem gives us a conservation law which we will then use to obtain the two desired properties. In this case we further find that the $\rho (a)$ used by Hélein will be explicitly of the form $\rho (a)(q)=\eta _a(q)$ for $\eta _a$ some fixed anti-symmetric matrix independent of *q*, which will considerably ease computations.

In particular, we obtain the following.

### Theorem 1.2

(Conservation law for $\varepsilon$-harmonic maps) Let (*M*, *g*) be a Riemannian surface, $(N,h)\hookrightarrow \mathbb {R}^l$ be a homogeneous space isometrically and equivariantly embedded into Euclidean space with $\Pi :G\rightarrow O(l)$ the associated Lie group embedding. $u \in C^\infty (M,N)$ is a solution of (4) if and only if, for any $\eta$ of the form $\eta = \frac{\partial }{\partial t}\Pi (\gamma (t)) \big |_{t=0}$ for some $\gamma (t)$ a smooth path in *G* with $\gamma (0)$ the identity, we have

$$\begin{aligned} {{\,\textrm{div}\,}}_g \big ( \langle du, \eta u \rangle - \varepsilon d (\langle \Delta _g u , \eta u \rangle )+ 2\varepsilon \langle \Delta _g u, \eta du \rangle \big ) =0. \end{aligned}$$

### Remark 1.3

In the case of a harmonic map, we have $\varepsilon =0$, this then allows us to rewrite Hélein’s conservation law from [4] as

$$\begin{aligned} {{\,\textrm{div}\,}}_g\langle du,\eta u\rangle = \langle du,\eta du\rangle + \langle \Delta _g u,\eta u\rangle =0 \end{aligned}$$

in the case the target is equivariantly embedded into Euclidean space.

The following theorem was proved by Lamm in [8].

### Theorem 1.4

(Bubbling for $\varepsilon$-harmonic maps) Let $(M^2,g)$ be a smooth, compact Riemannian surface without boundary and let $(N^n,h)\hookrightarrow \mathbb {R}^l$ be a compact Riemannian manifold isometrically embedded into Euclidean space. Further, $u_\varepsilon \in C^\infty (M,N) \; (\varepsilon \rightarrow 0)$ is a collection of critical points of $\tilde{E}_\varepsilon$ with uniformly bounded $\varepsilon$-energy.

Then there exists a sequence with $\varepsilon _k \rightarrow 0$ and at most finitely many points $x_1,...,x_p \in M$ such that

$$\begin{aligned} u_{\varepsilon _k}&\rightharpoonup u_0 \text { in } W^{1,2}(M,N), \\ u_{\varepsilon _k}&\rightarrow u_0 \text { in } C_{loc}^m(M \setminus \{x_1,...,x_p \}) \; \forall m \in \mathbb {N}, \end{aligned}$$

where $u_0 \in C^\infty (M,N)$ is a smooth harmonic map.

Further performing a blow-up at each $x_i, 1\le i \le p$, there exist at most finitely many non-trivial smooth harmonic maps $\omega ^{i,j}: S^2 \rightarrow N, 1\le j \le j_i$, sequences of points $x_k^{i,j}\in M, x_k^{i,j} \rightarrow x_i$, and sequences of radii $t_k^{i,j} \in \mathbb {R}_+$, $t_k^{i,j}\rightarrow 0$, such that

$$\begin{aligned} \max \Bigg \{ \frac{t_k^{i,j}}{t_k^{i,j'}} , \frac{t_k^{i,j'}}{t_k^{i,j}}, \frac{\text {dist}(x_k^{i,j}, x_k^{i,j'})}{t_k^{i,j}+ t_k^{i,j'}} \Bigg \}&\rightarrow \infty \quad \forall 1 \le i \le p, \; 1 \le j,j' \le j_i, \; j \ne j', \nonumber \\ \frac{\varepsilon _k}{(t_{k}^{i,j})^2}&\rightarrow 0 \quad \;\; \forall 1 \le i \le p, \; 1 \le j \le j_i. \end{aligned}$$

We will prove the following.

### Theorem 1.5

(Energy identity and no neck property for $\varepsilon$-harmonic maps) Under the conditions of Theorem 1.4 with the additional assumption that $(N^n,h) \hookrightarrow \mathbb {R}^l$ is a homogeneous space isometrically and equivariantly embedded into Euclidean space, we have the following.

- **The energy identity**
  $$\begin{aligned} \lim _{k\rightarrow \infty } \tilde{E}_{\varepsilon _k}[u_{\varepsilon _k}] = E_0[u_0] + \sum \limits _{i=1}^p \sum \limits _{j=1}^{j_i} E_0[\omega ^{i,j}] \end{aligned}$$
  and
  $$\begin{aligned} \int \limits _{B_{R_0}(x_i)} \big ( \vert \nabla \omega _k^{j_i} \vert ^2 + \varepsilon _k \vert \Delta \omega _k^{j_i} \vert ^2 \big ) \rightarrow \int \limits _{B_{R_0}(x_i)} \vert \nabla u_0 \vert ^2, \quad \forall 1 \le i \le p, \end{aligned}$$
  where $\omega _k^{j_i}= u_{\varepsilon _k}- \sum \limits _{j=1}^{j_i} (\omega ^{i,j}(\frac{\cdot - x_k^{i,j}}{t_k^{i,j}})- \omega ^{i,j}(\infty ))$ and $0<R_0 < \frac{1}{2} \min \big \{ \text {inj}(M),$ $\min \{ \text {dist}(x_i,x_j) \;|\; 1\le i \ne j \le p\} \big \}$ is some number and $\infty$ is identified with the north pole of $S^2$ by stereographic projection.
- **The no-neck property**
  $$\begin{aligned} \lim _{k \rightarrow 0} \Big \Vert u_{\varepsilon _k}(\cdot ) - u_0(\cdot ) - \sum \limits _{i=1}^p \sum \limits _{j=1}^{j_i} \big [\omega ^{i,j} \big (\frac{\cdot - x_j^{i,j}}{t_k^{i,j}} \big ) - \omega ^{i,j}(\infty )\big ] \Big \Vert _{L^\infty } = 0. \end{aligned}$$

The following theorem was proved by Sacks and Uhlenbeck in [19].

### Theorem 1.6

(Bubbling for $\alpha$-harmonic maps) Let $(M^2,g)$ be a smooth, compact Riemannian surface without boundary and let $(N^n,h)\hookrightarrow \mathbb {R}^l$ be a compact Riemannian manifold isometrically embedded into Euclidean space. Further, $u_\alpha \in C^\infty (M,N)$ $(\alpha \rightarrow 1)$ is a collection of critical points of $\bar{E}_\alpha$ with uniformly bounded $\alpha$-energy.

Then there exists a sequence for $\alpha _k \rightarrow 1$ and at most finitely many points $x_1,...,x_p \in M$ such that

$$\begin{aligned} u_{\alpha _k}&\rightharpoonup u_0 \text { in } W^{1,2}(M,N) \\ u_{\alpha _k}&\rightarrow u_0 \text { in } C_{loc}^m(M \setminus \{x_1,...,x_p \}) \; \forall m \in \mathbb {N}, \end{aligned}$$

where $u_0 \in C^\infty (M,N)$ is a smooth harmonic map.

Further performing a blow-up at each $x_i, 1\le i \le p$, there exist at most finitely many non-trivial smooth harmonic maps $\omega ^{i,j}: S^2 \rightarrow N, 1\le j \le j_i$, sequences of points $x_k^{i,j}\in M, x_k^{i,j} \rightarrow x_i$, and sequences of radii $t_k^{i,j} \in \mathbb {R}_+$, $t_k^{i,j}\rightarrow 0$, such that

$$\begin{aligned} \max \Big \{ \frac{t_k^{i,j}}{t_k^{i,j'}} , \frac{t_k^{i,j'}}{t_k^{i,j}}, \frac{\text {dist}(x_k^{i,j}, x_k^{i,j'})}{t_k^{i,j}+ t_k^{i,j'}} \Big \}&\rightarrow \infty , \quad \forall 1 \le i \le p, \; 1 \le j,j' \le j_i, \; j \ne j'. \nonumber \\ \end{aligned}$$

We will prove the following.

### Theorem 1.7

(Energy identity and no neck property for $\alpha$-harmonic maps) Under the conditions of Theorem 1.6 and the further assumption that *N* is a homogeneous manifold, we have the following:

- **The energy identity**
  $$\begin{aligned} \lim _{k\rightarrow \infty } \bar{E}_{\alpha _k}[u_{\alpha _k}] = E_0[u_0] + \sum \limits _{i=1}^p \sum \limits _{j=1}^{j_i} E_0[\omega ^{i,j}] \end{aligned}$$
  and
  $$\begin{aligned} \int \limits _{B_{R_0}(x_i)} \big ( (1+ \vert \nabla \omega _k^{j_i} \vert ^2)^{\alpha _k}-1 \big ) \rightarrow \int \limits _{B_{R_0}(x_i)} \vert \nabla u_0 \vert ^2, \quad \forall 1 \le i \le p, \end{aligned}$$
  where $\omega _k^{j_i}= u_{\alpha _k}- \sum \limits _{j=1}^{j_i} \big (\omega ^{i,j}(\frac{\cdot - x_k^{i,j}}{t_k^{i,j}})- \omega ^{i,j}(\infty )\big )$ and $0<R_0 < \frac{1}{2} \min \{ \text {inj}(M),$ $\min \{ \text {dist}(x_i,x_j) \; |\; 1\le i \ne j \le p\} \}$ is some number and $\infty$ is identified with the north pole of $S^2$ by stereographic projection.
- **The no-neck property**
  $$\begin{aligned} \lim _{k \rightarrow \infty } \Big \Vert u_{\alpha _k}(\cdot ) - u_0(\cdot ) - \sum \limits _{i=1}^p \sum \limits _{j=1}^{j_i} \big [\omega ^{i,j} \big (\frac{\cdot - x_j^{i,j}}{t_k^{i,j}} \big ) - \omega ^{i,j}(\infty ) \big ] \Big \Vert _{L^\infty } = 0. \end{aligned}$$

Finally, Li and Wang constructed in [15] a general target manifold such that the energy identity does not hold in the case of $\alpha$-harmonic maps. By following this construction, we show that their manifold also gives a counterexample for the energy identity in the case of $\varepsilon$-harmonic maps. Though we note that this construction relies on varying homotopy classes and so does not say anything of the general case for a fixed homotopy class.

## Energy bound for $\varepsilon$-harmonic maps

In this section, we prove an $L^2$-energy bound for $\varepsilon$-harmonic maps in the neck region. The key fact lies in the construction of the conservation law, Theorem 1.2. We will then use this along with the estimates obtained by Lamm in [8] to obtain the energy bound. We also note that the conservation law is of a form similar to the one obtained by Lamm for the spherical case, and one can use his approach to obtain the energy bound in the homogeneous case. We, however, present a different proof, as from our proof the no neck condition will easily follow. We also note again that this proof will only work in the case that our target is equivariantly embedded.

To prove Theorem 1.2 we first need the following lemma.

### Lemma 2.1

Let $(N,h)\hookrightarrow \mathbb {R}^l$ be a homogeneous space isometrically and equivariantly embedded into Euclidean space with $\Pi :G\rightarrow O(l)$ the associated Lie group embedding. Then there exists a finite collection $(\gamma _i)_{i=1}^I$ of smooth paths in G with $\gamma _i(0)=\text {id}$ such that, setting $\eta _i=\frac{\partial }{\partial t}(\Pi (\gamma _i(t)))\big |_0$, the collection $(\eta _iq)_{i=1}^I$ spans $T_qN$ for any $q\in N$.

As our $\eta$ are the same objects as Hélein’s $\rho$, the $\eta$ span for the same reason as the $\rho$ do.

### Proof

Take some basis $a_1,...,a_I$ of $\mathfrak {g}$, the Lie algebra of *G*, and paths $\gamma _i(t)$ in *G* with $\gamma _i(0)=$id and $d\gamma _i|_0=a_i$ for $i=1,...,I$. Now set $\eta _i=\frac{\partial }{\partial t}(\Pi (\gamma _i))|_0$. The $\eta _i q$ will now span the tangent space for each *q* due to the transitivity of the action of *G*. Explicitly given $q\in N$ and $\omega \in T_qN$ we can find some path $\zeta$ in *N* with $\zeta (0)=q$ and $\zeta '(0)=\omega$. This can then be lifted to some path $\gamma$ in *G* with $\gamma (0)=\text {id}$ and $\Pi (\gamma (t))q=\zeta (t)$. Now $\omega =\frac{\partial }{\partial t}\Pi (\gamma (t))|_0=d\Pi |_{\text {id}}(d\gamma |_0)$. We know that $d\gamma |_0$ is in the span of the $a_i$ so $\omega$ must be in the span of the $\eta _iq$. $\square$

### Proof of Theorem 1.2

First, to motivate our proof, one can observe that $\tilde{E}_\varepsilon$ is invariant under $\Pi (G)\subset O(l)$

$$\begin{aligned} \tilde{E}_\varepsilon [\Pi (\gamma (t))\circ u] = \tilde{E}_\varepsilon [u] \end{aligned}$$

for all *t*, so we should have some conservation law by Noether’s theorem. Now explicitly for any $q\in N$ we have that $\Pi (\gamma (t))q$ is some path in *N* so $\eta q\in T_qN$. But also $\Pi (\gamma (t))$ is a path in *O*(*l*) so $\eta$ is in $\mathfrak {o}(l)$, in particular it can be viewed as an anti-symmetric matrix. Further, *u* being a solution of (4) implies that $(\Delta _g u - \varepsilon \Delta ^2_gu) \in (T_uN)^\perp$, where $\Delta _g$ is the Laplacian with respect to the metric on *M*. We can then observe, noting that $\eta$ is constant,

5 $$\begin{aligned} \begin{aligned}&0= \langle \Delta _g u - \varepsilon \Delta _g^2 u , \eta u \rangle \\&= {{\,\textrm{div}\,}}_g \big ( \langle du, \eta u \rangle - \varepsilon d \langle \Delta _g u , \eta u \rangle + 2\varepsilon \langle \Delta _g u, \eta du \rangle \big ) \\&\quad -\langle du, \eta du \rangle - \varepsilon \langle \Delta _g u, \eta \Delta _g u \rangle . \end{aligned} \end{aligned}$$

The last two terms are zero by anti-symmetry of $\eta$ and so the conservation law holds for any fixed $\eta$.

For the only if part of the statement we note that if the conservation law holds for any $\eta$ then, by reversing the steps in (5), we must have

$$\begin{aligned} 0 = \langle \Delta _g u - \varepsilon \Delta _g^2 u , \eta u \rangle \end{aligned}$$

for any $\eta$. By Lemma 2.1 the possible $\eta u$ span $T_uN$, so we must have $(\Delta _g u - \varepsilon \Delta ^2_gu) \in (T_uN)^\perp$ and so *u* is $\varepsilon$-harmonic.

Note that if we chose conformal coordinates with factor $e^{2\lambda }$ then the conservation law locally becomes

6 $$\begin{aligned} {{\,\textrm{div}\,}}\big ( \langle du, \eta u \rangle - \varepsilon d (e^{-2\lambda }\langle \Delta u , \eta u \rangle )+ 2\varepsilon e^{-2\lambda }\langle \Delta u, \eta du \rangle \big ) =0 . \end{aligned}$$

In [8] Lamm showed that these $\varepsilon$-harmonic maps indeed undergo a bubbling procedure, as we will outline.

### Lemma 2.2

Given a sequence $\varepsilon _k\rightarrow 0$ and $u_{\varepsilon _k}$ $\varepsilon _k$-harmonic maps with uniformly bounded $\varepsilon _k$-energy. Then there exists some subsequence (which we will immediately relabel $\varepsilon _k$), $u_0\in C^\infty (M,N)$ harmonic, and some finite set $\Sigma$ such that

$$\begin{aligned} u_{\varepsilon _k}\rightarrow u_0 \quad in \quad C^m_{\text {loc}}(M\backslash \Sigma ,N). \end{aligned}$$

Using a standard induction argument, as in [10], we may also assume that there is only one bubble, $\Sigma =\{x_0\}$. We then have the following.

### Lemma 2.3

(Lemma 3.1 [8]) There exist $t_k\in \mathbb {R}^+$, $t_k\rightarrow 0$, $x_k\in M$, $x_k\rightarrow x_0$ and a non-trivial, smooth harmonic map $\omega :S^2\rightarrow N$ such that

$$\begin{aligned} \frac{\varepsilon _k}{t_k^2}\rightarrow 0, \end{aligned}$$

$$\begin{aligned} u_{\varepsilon _k}(x_k+t_k\cdot )\rightarrow \omega \;\; in \quad C^m_{\text {loc}}(\mathbb {R}^2,N) \quad \forall m \in \mathbb {N}. \end{aligned}$$

In this case, the energy identity

$$\begin{aligned} \lim _{k\rightarrow \infty }\tilde{E}_{\varepsilon _k}[u_{\varepsilon _k}]=E_0[u_0]+E_0[\omega ] \end{aligned}$$

is equivalent to having

7 $$\begin{aligned} \tilde{E}_{\varepsilon _k}[u_{\varepsilon _k};B_{R_0}(x_k)\backslash B_{Rt_k}(x_k)]\rightarrow 0 \end{aligned}$$

as $$k\rightarrow \infty ,R\rightarrow \infty ,R_0\rightarrow 0$$.

We will now recall some more results on $\varepsilon$-harmonic maps from [8]. First, we have a regularity result.

### Lemma 2.4

(Corollary 2.10 [8]) There exist $\delta _0>0$ and $c>0$ such that if $u_\varepsilon \in C^\infty (M,N)$ is $\varepsilon$-harmonic such that for some $x_0\in M, R>0$ we have $\tilde{E}_\varepsilon (u_\varepsilon ,B_{32R}(x_0))<\delta _0$. Then for all $\varepsilon >0$ sufficiently small and any $k\in \mathbb {N}$

$$\begin{aligned} \sum _{i=1}^kR^i||\nabla ^iu_\varepsilon ||_{L^\infty (B_R(x_0))}\le c\sqrt{\tilde{E}_\varepsilon (u_\varepsilon ,B_{32R}(x_0))}. \end{aligned}$$

Now fix $0<\delta <\delta _0$. Then, as a consequence of there only existing one bubble, there exist $R_0>0$ small enough, $R>0$ large enough, and $k_1>0$ large enough such that for all $k\ge k_1$ we have

$$\begin{aligned} \tilde{E}_{\varepsilon _k}[u_{\varepsilon _k}; B_{2r}\setminus B_r] < \delta \end{aligned}$$

for every $r\in [Rt_k, \frac{R_0}{2}]$. Combining these facts gives

8 $$\begin{aligned} \sum _{i=1}^4 \vert x\vert ^i \vert \nabla ^i u_{\varepsilon _k} \vert (x) \le c \sqrt{\delta } \quad \quad \forall \; 2Rt_k \le \vert x \vert \le \frac{R_0}{4}. \end{aligned}$$

This immediately gives us a bound for the $L^{2,\infty }$-norm

$$\begin{aligned} \Vert \nabla u_{\varepsilon _k} \Vert _{L^{2,\infty }(B_{\frac{R_0}{4}} \setminus B_{2Rt_k})} \le c\sqrt{\delta } \; \Big \Vert \frac{1}{\vert x \vert } \Big \Vert _{L^{2,\infty }(\mathbb {R}^2)} \le c\sqrt{\delta }. \end{aligned}$$

It will now be sufficient to find a uniform bound on the $L^{2,1}$-norm of $\nabla u_{\varepsilon _k}$. $L^{2,\infty }$ is the dual space of $L^{2,1}$, so if we have $\Vert \nabla u_{\varepsilon _k} \Vert _{L^{2,1}(B_{\frac{R_0}{8}} \setminus B_{4Rt_k}, N)}\le c$, we can conclude

9 $$\begin{aligned} E_0[u_{\varepsilon _k}; B_{\frac{R_0}{8}}\setminus B_{4Rt_k}]&\le c \Vert \nabla u_{\varepsilon _k} \Vert _{L^{2,1}(B_{\frac{R_0}{8}} \setminus B_{4Rt_k}, N)} \cdot \Vert \nabla u_{\varepsilon _k} \Vert _{L^{2,\infty }(B_{\frac{R_0}{8}} \setminus B_{4Rt_k},N)} \nonumber \\&\rightarrow 0. \end{aligned}$$

The $\varepsilon _k$-term of the energy will also vanish on this annulus, as

10 $$\begin{aligned} \varepsilon _k \int _{B_{\frac{R_0}{4}} \setminus B_{2Rt_k}} \vert \Delta u_{\varepsilon _k} \vert ^2 \le c \delta \varepsilon _k \int _{B_{\frac{R_0}{4}} \setminus B_{2Rt_k}} \frac{1}{\vert x \vert ^4} \le \frac{c\delta }{R^2}\frac{\varepsilon _k}{t_k^2} \rightarrow 0 \end{aligned}$$

using Lemma 2.3 and (8). Combining (9) and (10) shows that

$$\begin{aligned} \tilde{E}_{\varepsilon _k}[u_{\varepsilon _k}; B_{\frac{R_0}{8}} \setminus B_{4Rt_k}] \rightarrow 0 . \end{aligned}$$

So, it remains to show that the $L^{2,1}$ norm for the gradient of $u_{\varepsilon _k}$ is bounded on this annulus.

We first mention two results that will be used to bound this $L^{2,1}$-norm.

### Lemma 2.5

(Wente inequality, [2, 20]) If $f,g\in W^{1,2}(B)$ and *u* is the unique solution in $W^{1,2}_0(B)$ to $\Delta u=\nabla f\nabla ^\bot g$, then $\Delta u$ is in $\mathcal {H}^1{(\mathbb {R}^2)}$ with

$$\begin{aligned} ||\nabla u||_{L^{2,1}}\le c||\nabla f\nabla ^\bot g||_{\mathcal {H}^1}\le c||\nabla f||_{L^2}||\nabla g||_{L^2}. \end{aligned}$$

### Lemma 2.6

(Lemma 2.4, [13]) If $F,u\in C_c^\infty (\mathbb {R}^2)$ satisfy $\Delta u={{\,\textrm{div}\,}}(F)$, then

$$\begin{aligned} ||\nabla u||_{L^{2,1}}\le c||F||_{L^{2,1}} . \end{aligned}$$

Now we start by constructing an estimate of $u_{\varepsilon _k}$ as follows.

11 $$\begin{aligned} \tilde{u}_{\varepsilon _k}(x)= \varphi _{\frac{R_0}{16}}(x) \big ( (1-\varphi _{4R t_k}(x)) (u_{\varepsilon _k}(x)- \bar{u}_{\varepsilon _k}^2) + \bar{u}_{\varepsilon _k}^2- \bar{u}_{\varepsilon _k}^1 \big ), \end{aligned}$$

where we have fixed some $\varphi \in C_c^\infty (B_2)$ such that $\varphi =1$ on $B_1$, taken $\varphi _t(x)= \varphi (\frac{x}{t})$ and have set

$$\begin{aligned} \bar{u}_{\varepsilon _k}^1&= \frac{1}{|B_{\frac{R_0}{8}} \setminus B_{\frac{R_0}{16}}|} \int \limits _{B_{\frac{R_0}{8}} \setminus B_{\frac{R_0}{16}}} u_{\varepsilon _k}(x) \; dx, \\ \bar{u}_{\varepsilon _k}^2&= \frac{1}{|B_{8R t_k} \setminus B_{4R t_k }|} \int \limits _{B_{8R t_k} \setminus B_{4R t_k}} u_{\varepsilon _k}(x) \; dx . \end{aligned}$$

Then one can calculate

12 $$\begin{aligned} \nabla \tilde{u}_{\varepsilon _k}&=\varphi _{\frac{R_0}{16}} (1-\varphi _{4R t_k})\nabla u_{\varepsilon _k} + \nabla (\varphi _{\frac{R_0}{16}}) ( u_{\varepsilon _k} - \bar{u}_{\varepsilon _k}^1) \nonumber \\&\quad \quad -\nabla (\varphi _{4R t_k}) ( u_{\varepsilon _k} - \bar{u}_{\varepsilon _k}^2). \end{aligned}$$

We note that the support of $\nabla \varphi _{\frac{R_0}{16}}$ is contained in $B_{\frac{R_0}{8}}\backslash B_{\frac{R_0}{16}}$. Then, using (8) to get a bound on $\nabla u_{\varepsilon _k}$, we get for *k* sufficiently large

13 $$\begin{aligned} |u_{\varepsilon _k}(x) - \bar{u}_{\varepsilon _k}^1|&=\frac{1}{|B_{\frac{R_0}{8}} \setminus B_{\frac{R_0}{16}}|} \int \limits _{B_{\frac{R_0}{8}} \setminus B_{\frac{R_0}{16}}} |u_{\varepsilon _k}(x)-u_{\varepsilon _k}(y)| \; dy \nonumber \\&\le c\sqrt{\delta } \end{aligned}$$

for $x\in B_{\frac{R_0}{8}} \setminus B_{\frac{R_0}{16}}$. Similarly, we have

14 $$\begin{aligned} |u_{\varepsilon _k}(x) - \bar{u}_{\varepsilon _k}^2|&= \frac{1}{|B_{8R t_k} \setminus B_{4R t_k }|} \int \limits _{B_{8R t_k} \setminus B_{4R t_k }} |u_{\varepsilon _k}(x) -u_{\varepsilon _k}(y)| \;dy \nonumber \\&\le c\sqrt{\delta } \end{aligned}$$

for $x\in B_{8Rt_k}\backslash B_{4Rt_k}$. This then gives

15 $$\begin{aligned} ||\nabla \tilde{u}_{\varepsilon _k}||_{L^2(\mathbb {R}^2)} \le ||\nabla u_{\varepsilon _k}||_{L^2(B_{\frac{R_0}{8}} \setminus B_{4Rt_k })}+c\sqrt{\delta } \end{aligned}$$

for *k* sufficiently large. Note that in two dimensions the $L^{2,1}$-norm of $\nabla \varphi _{\rho }$ is independent of $\rho$ and therefore bounded. This is easily checked by using

16 $$\begin{aligned} \big \vert \{x: \vert \nabla \varphi _\rho (x)\vert \ge t \} \big \vert ^{\frac{1}{2}} = \big \vert \{x: \big \vert (\nabla \varphi )\Big (\frac{x}{\rho }\Big )\big \vert \ge \rho t \}\big \vert ^{\frac{1}{2}} = \rho \big \vert \{x: \vert \nabla \varphi (x)\vert \ge \rho t \}\big \vert ^{\frac{1}{2}} \end{aligned}$$

and the $L^{2,1}$-norm definition.

We now note the fact that our conservation law (6) holds on $B_{R_0}$ so there exists $L_k\in C^\infty ({B_{R_0}})$ with

17 $$\begin{aligned} \nabla ^\perp L_k = \langle \nabla u_{\varepsilon _k}, \eta u_{\varepsilon _k} \rangle - \varepsilon _k \nabla \langle e^{-2\lambda }\Delta u_{\varepsilon _k} , \eta u_{\varepsilon _k} \rangle + 2\varepsilon _k \langle e^{-2\lambda } \Delta u_{\varepsilon _k}, \eta \nabla u_{\varepsilon _k} \rangle . \end{aligned}$$

Now by the Hodge decomposition there exist $P_k,Q_k\in C^\infty _c({\mathbb {R}^2})$ with

18 $$\begin{aligned} \langle \nabla \tilde{u}_{\varepsilon _k}, \eta u_{\varepsilon _k} \rangle = \nabla P_k +\nabla ^\perp Q_k. \end{aligned}$$

Taking the curl of (18) gives the div-curl relationship

$$\begin{aligned} \Delta Q_k&= \text {curl}\langle \nabla \tilde{u}_{\varepsilon _k}, \eta u_{\varepsilon _k} \rangle \nonumber \\&= \langle \nabla \tilde{u}_{\varepsilon _k}, \nabla ^\perp ( \eta u_{\varepsilon _k}) \rangle . \end{aligned}$$

Using the same construction as (11) on a larger annulus we can construct $\hat{u}_{\varepsilon _k}\in C_c^\infty (\mathbb {R}^2,\mathbb {R}^l)$ such that

$$\begin{aligned} \nabla \hat{u}_{\varepsilon _k}&=\nabla u_{\varepsilon _k} \quad \text { on }B_{\frac{R_0}{8}}\backslash B_{4Rt_k},\\ ||\nabla \hat{u}_{\varepsilon _k}||_{L^2(\mathbb {R}^2)}&\le c||\nabla u_{\varepsilon _k}||_{L^2(B_{\frac{R_0}{4}}\backslash B_{2Rt_k})}+c\sqrt{\delta }\le c \end{aligned}$$

using the uniform bound on $\varepsilon$-energy for the final inequality. Then $\Delta Q=\langle \nabla \tilde{u}_{\varepsilon _k}, \nabla ^\perp (\eta \hat{u}_{\varepsilon _k} )\rangle$ and so using (15) we get

19 $$\begin{aligned} \nonumber ||\nabla Q_k||_{L^{2,1}{(\mathbb {R}^2)}}\le&c||\nabla \tilde{u}_{\varepsilon _k}||_{L^2(\mathbb {R}^2)}\cdot ||\nabla (\eta \hat{u}_{\varepsilon _k})||_{L^2(\mathbb {R}^2)}\\ \le&c||\nabla u_{\varepsilon _k}||_{L^2(B_{\frac{R_0}{8}} \setminus B_{4Rt_k })} + c\sqrt{\delta }. \end{aligned}$$

Now take the divergence of (18) and use (12) to get

$$\begin{aligned} \Delta P_k&= {{\,\textrm{div}\,}}\langle \nabla \tilde{u}_{\varepsilon _k}, \eta u_{\varepsilon _k} \rangle \nonumber \\&= {{\,\textrm{div}\,}}\Big ( \varphi _{\frac{R_0}{16}} (1-\varphi _{4R t_k})\big ( \nabla ^\perp L_k \nonumber +\varepsilon _k \nabla \langle e^{-2\lambda }\Delta u_{\varepsilon _k} , \eta u_{\varepsilon _k} \rangle \\&\qquad \qquad \qquad \qquad \qquad \quad - 2\varepsilon _ke^{-2\lambda } \langle \Delta u_{\varepsilon _k}, \nabla (\eta u_{\varepsilon _k}) \rangle \big ) \nonumber \\&\quad + \nabla (\varphi _{\frac{R_0}{16}}) \langle u_{\varepsilon _k} -\bar{u}_{\varepsilon _k}^1,\eta u_{\varepsilon _k} \rangle -\nabla (\varphi _{4R t_k}) \langle u_{\varepsilon _k} - \bar{u}_{\varepsilon _k}^2,\eta u_{\varepsilon _k} \rangle \Big ). \end{aligned}$$

Now breaking up the terms we define $(\Phi _i)_{i=1,2,3}$ to be the unique solutions in $C^\infty _c({\mathbb {R}^2})$ of

20 $$\begin{aligned} \Delta \Phi _1=&{{\,\textrm{div}\,}}\big ( \varphi _{\frac{R_0}{16}} (1-\varphi _{4R t_k}) \nabla ^\bot L_k\big ), \end{aligned}$$

21 $$\begin{aligned} \Delta \Phi _2=&\varepsilon _k{{\,\textrm{div}\,}}\big ( \varphi _{\frac{R_0}{16}} (1-\varphi _{4R t_k})\big ( \nabla \langle e^{-2\lambda }\Delta u_{\varepsilon _k} , \eta u_{\varepsilon _k} \rangle \nonumber \\&\hspace{12mm}- 2e^{-2\lambda } \langle \Delta u_{\varepsilon _k}, \eta \nabla u_{\varepsilon _k} \rangle \big ) \big ),\end{aligned}$$

22 $$\begin{aligned} \Delta \Phi _3=&{{\,\textrm{div}\,}}\big ( \nabla (\varphi _{\frac{R_0}{16}}) \langle u_{\varepsilon _k} - \bar{u}_{\varepsilon _k}^1,\eta u_{\varepsilon _k} \rangle \nonumber \\&\hspace{9mm} -\nabla (\varphi _{4R t_k}) \langle u_{\varepsilon _k} - \bar{u}_{\varepsilon _k}^2,\eta u_{\varepsilon _k} \rangle \big ). \end{aligned}$$

We can now bound these all individually, first we have

23 $$\begin{aligned} \Delta \Phi _1= \nabla (\varphi _{\frac{R_0}{16}} (1-\varphi _{t_k R})) \nabla ^\perp L_k \end{aligned}$$

giving us a div-curl relationship. We define $\tilde{L}_k$ similarly to $\tilde{u}$ in (11) by

$$\begin{aligned} \nabla \tilde{L}_{k}(x)&=\varphi _{\frac{R_0}{8}}(x) (1-\varphi _{2R t_k }(x))\nabla L_k(x) + \nabla (\varphi _{\frac{R_0}{8}}(x)) ( L_{k}(x) - \bar{L}_{k}^1) \nonumber \\&\quad \quad -\nabla (\varphi _{2R t_k }(x)) ( L_k(x) - \bar{L}_{k}^2) \end{aligned}$$

where, similarly to the construction of $\tilde{u}_{\varepsilon _k}$, we set $\bar{L}_{k}^1,\bar{L}_{k}^2$ to be the mean of $L_k$ on $B_{\frac{R_0}{4}} \setminus B_{\frac{R_0}{8}}$ and $B_{4Rt_k} \setminus B_{2Rt_k}$ respectively. This gives us for *k* sufficiently large

24 $$\begin{aligned} \nabla \tilde{L}_{k}&=\nabla {L}_{k} \quad on \quad B_{\frac{R_0}{8}}\backslash B_{4Rt_k}, \nonumber \\ ||\nabla \tilde{L}_{k}||_{L^2(\mathbb {R}^2)}&\le c||\nabla {L}_{k}||_{L^2(B_{\frac{R_0}{4}}\backslash B_{2Rt_k})}+c\sqrt{\delta }. \end{aligned}$$

Where we used (8), (17), Lemma 2.3 and the fact that $||e^{-2\lambda (x)}||_\infty$ and $||De^{-2\lambda (x)}||_\infty$ are uniformly bounded to get $|\nabla {L}_{k}(x)|\le \frac{c\sqrt{\delta }}{|x|}$ on $B_{\frac{R_0}{4}}\backslash B_{2Rt_k}$ for *k* large enough. The inequality then follows analogously to $(15)$. We also note, using Lemma 2.3 and (17), that

25 $$\begin{aligned} ||\nabla L_k||_{L^2{(B_{\frac{R_0}{4}}\backslash B_{2Rt_k})}}\le c||\nabla u_{\varepsilon _k}||_{L^2{(B_{\frac{R_0}{4}} \setminus B_{2Rt_k })}}+c\sqrt{\delta } \end{aligned}$$

for *k* large enough. Now using Lemma 2.5, (23), (24) and (25) gives

26 $$\begin{aligned} {\begin{matrix} & ||\nabla \Phi _1||_{L^{2,1}{(\mathbb {R}^2)}} \le ||\nabla (\varphi _{\frac{R_0}{16}}(x) (1-\varphi _{4Rt_k}(x)))||_{L^2{(\mathbb {R}^2)}}\cdot ||\nabla \tilde{L}_k||_{L^2{(\mathbb {R}^2)}}\\ & \le c ||\nabla L_k||_{L^2{(B_{\frac{R_0}{4}}\backslash B_{2Rt_k})}}+c\sqrt{\delta }\\ & \le c||\nabla u_{\varepsilon _k}||_{L^2{(B_{\frac{R_0}{4}} \setminus B_{2Rt_k })}}+c\sqrt{\delta } \end{matrix}} \end{aligned}$$

for *k* large enough.

For the $\Phi _2$ term we note that $\varphi _{\frac{R_0}{16}}(x) (1-\varphi _{4Rt_k}(x))\le 1$ and so using Lemma 2.6, $(8)$ and the bounds on the conformal factor gives

$$\begin{aligned} ||\nabla \Phi _2||_{L^{2,1}{(\mathbb {R}^2)}}&\le c\varepsilon _k|| \nabla (e^{-2\lambda }\langle \Delta \nonumber u_{\varepsilon _k} , \eta u_{\varepsilon _k} \rangle )- 2e^{-2\lambda } \langle \Delta u_{\varepsilon _k}, \eta \nabla u_{\varepsilon _k} \rangle ||_{L^{2,1}{(B_{\frac{R_0}{8}}\backslash B_{4Rt_k})}} \\&\le c\varepsilon _k \left\| \frac{1}{|x|^3}\right\| _{L^{2,1}{(B_{\frac{R_0}{8}}\backslash B_{4Rt_k})}}. \end{aligned}$$

Now we wish to calculate this Lorentz norm.

### Lemma 2.7

$\displaystyle \Big \Vert \frac{1}{|x|^3}\Big \Vert _{L^{2,1}{(B_\alpha \backslash B_\beta )}}= c\frac{1}{\beta ^2}\sqrt{1-\frac{\beta ^2}{\alpha ^2}}$ for some constant *c*.

### Proof

For ease of notation set $\displaystyle f(x)=\frac{1}{|x|^3}$. First compute the distribution function $\lambda _f$.

$$\begin{aligned} \lambda _f (s)= {\left\{ \begin{array}{ll} \pi (\alpha ^2-\beta ^2) &  \quad s< \frac{1}{\alpha ^3} \\ \pi (s^{-\frac{2}{3}}-\beta ^2) &  \quad \frac{1}{\alpha ^3}< s< \frac{1}{\beta ^3} \\ 0 &  \quad \frac{1}{\beta ^3} < s \\ \end{array}\right. } \end{aligned}$$

this then gives

$$\begin{aligned} f^*(t) = {\left\{ \begin{array}{ll} \big (\frac{t}{\pi }+\beta ^2\big )^{-\frac{3}{2}} &  \quad t< \pi (\alpha ^2-\beta ^2) \\ 0 &  \quad \pi (\alpha ^2-\beta ^2) < t .\\ \end{array}\right. } \end{aligned}$$

Finally

$$\begin{aligned} ||f||_{L^{2,1}(B_\alpha \backslash B_\beta )}&= \int \limits _0^{\pi (\alpha ^2-\beta ^2)} t^{-\frac{1}{2}} \big (\frac{t}{\pi }+\beta ^2\big )^{-\frac{3}{2}} \; dt \; = \; \frac{2\pi ^{\frac{1}{2}}}{\beta ^2}\bigg [\frac{t^{\frac{1}{2}}}{(t+\beta ^2)^{\frac{1}{2}}}\bigg ]^{\alpha ^2-\beta ^2}_0 \nonumber \\&=\frac{2\pi ^{\frac{1}{2}}}{\beta ^2}\sqrt{1-\frac{\beta ^2}{\alpha ^2}}. \end{aligned}$$

$$\square $$

So using Lemma 2.3 we get $\lim \limits _{k\rightarrow \infty }||\nabla \Phi _2||_{L^{2,1}{(\mathbb {R}^2)}}=0$. In particular we may assume

27 $$\begin{aligned} ||\nabla \Phi _2||_{L^{2,1}{(\mathbb {R}^2)}} \le \sqrt{\delta } \end{aligned}$$

for *k* large enough.

Finally, we bound the $\Phi _3$ term. Note that Lemma 2.6 along with (13), (14) and (16) implies

28 $$\begin{aligned} ||\nabla \Phi _3||_{L^{2,1}{(\mathbb {R}^2)}}&\le \Vert \langle (u_{\varepsilon _k}- \bar{u}_{\varepsilon _k}^1), \eta u_{\varepsilon _k}\rangle \nabla \varphi _{\frac{R_0}{16}} \Vert _{L^{2,1}(\mathbb {R}^2)} \nonumber \\&\quad + \Vert \langle (u_{\varepsilon _k}- \bar{u}_{\varepsilon _k}^2), \eta u_{\varepsilon _k}\rangle \nabla \varphi _{4Rt_k } \Vert _{L^{2,1}(\mathbb {R}^2)} \nonumber \\&\le c \sqrt{\delta } \big ( \Vert \nabla \varphi _{\frac{R_0}{16}} \Vert _{L^{2,1}(\mathbb {R}^2)}+ \Vert \nabla \varphi _{4R t_k } \Vert _{L^{2,1}(\mathbb {R}^2)} \big )\nonumber \\&\le c\sqrt{\delta }. \end{aligned}$$

Now as we have $\Delta P_k=\Delta \Phi _1+\Delta \Phi _2+\Delta \Phi _3$, and all functions are compactly supported, we must have $P_k=\Phi _1+\Phi _2+\Phi _3$. This then gives

29 $$\begin{aligned} ||\nabla P_k||_{L^{2,1}(\mathbb {R}^2)}\le ||\nabla \Phi _1||_{L^{2,1}(\mathbb {R}^2)}+||\nabla \Phi _2||_{L^{2,1}(\mathbb {R}^2)}+||\nabla \Phi _3||_{L^{2,1}(\mathbb {R}^2)}. \end{aligned}$$

So overall using the Hodge decomposition (18), and the equations (19), (26), (27), (28) and (29) we achieve, for *k* large enough

30 $$\begin{aligned} ||\langle \nabla \tilde{u}_{\varepsilon _k}, \eta u_{\varepsilon _k} \rangle ||_{L^{2,1}(\mathbb {R}^2)} \le&||\nabla P_k||_{L^{2,1}(\mathbb {R}^2)} + ||\nabla Q_k||_{L^{2,1}(\mathbb {R}^2)}\nonumber \\ \le&c||\nabla u_{\varepsilon _k}||_{L^2(B_{\frac{R_0}{4}} \setminus B_{2Rt_k }))}+c\sqrt{\delta }. \end{aligned}$$

Noting here that the constant *c* potentially depends on $\eta$.

Now using the definition of $\tilde{u}_{\varepsilon _k}$, (11), we note that on $B_{\frac{R_0}{16}}\backslash B_{8Rt_k}$ we have $\nabla \tilde{u}_{\varepsilon _k}(x)=\nabla u_{\varepsilon _k}(x)$, which by definition always lies in $T_{u_{\varepsilon _k}(x)}N$. Using Lemma 2.1 we may take some finite spanning collection of $\eta _i$, which then gives

31 $$\begin{aligned} |\nabla u_{\varepsilon _k}|\le c\sum _i|\langle \nabla \tilde{u}_{\varepsilon _k}, \eta _i u_{\varepsilon _k} \rangle |\quad \text {on}\;B_{\frac{R_0}{16}}\backslash B_{8Rt_k}. \end{aligned}$$

So, using the assumption that the $\varepsilon$-energy is uniformly bonded, (30) and (31) we get

32 $$\begin{aligned} || \nabla u_{\varepsilon _k}||_{L^{2,1}(B_{\frac{R_0}{16}}\backslash B_{8Rt_k})}&\le c\sum _i||\langle \nabla \tilde{u}_{\varepsilon _k}, \eta _i u_{\varepsilon _k} \rangle ||_{L^{2,1}(B_{\frac{R_0}{16}}\backslash B_{8Rt_k})}\\\nonumber&\le c\sum _i||\langle \nabla \tilde{u}_{\varepsilon _k}, \eta _i u_{\varepsilon _k} \rangle ||_{L^{2,1}(\mathbb {R}^2)}\\\nonumber&\le c||\nabla u_{\varepsilon _k}||_{L^2(B_{\frac{R_0}{4}} \setminus B_{2Rt_k }))}+c\sqrt{\delta }\\\nonumber&\le c \end{aligned}$$

which completes the energy bound on the annulus by our prior discussion.

## No neck property for $\varepsilon$-harmonic maps

The no-neck property will now follow easily from the energy identity. Because of the assumption of there only being one bubble, as in [13], all we need to prove is that

33 $$\begin{aligned} \lim _{\varepsilon _k \rightarrow 0} \big \Vert u_{\varepsilon _k}(\cdot ) - u_0(\cdot ) - \big ( \omega \Big (\frac{\cdot - x_k}{t_k} \Big ) - \omega (\infty ) \big ) \big \Vert _{L^\infty } = 0. \end{aligned}$$

This then reduces to showing that there is no oscillation on the neck region, specifically we need

$$\begin{aligned} \lim _{R_0 \rightarrow 0}\lim _{R \rightarrow \infty }\lim _{k \rightarrow \infty } \sup _{x,y \in B_{R_0}\setminus B_{R t_k}}\vert u_{\varepsilon _k}(x) - u_{\varepsilon _k}(y) \vert = 0. \end{aligned}$$

Then we note that, for $\tilde{u}_{\varepsilon _k}$ defined in (11),

$$\begin{aligned} \sup _{x,y \in B_{\frac{R_0}{16}}\setminus B_{8R t_k}} \vert u_{\varepsilon _k}(x) - u_{\varepsilon _k}(y) \vert&\le \sup _{x,y \in \mathbb {R}^2} \vert \tilde{u}_{\varepsilon _k}(x) - \tilde{u}_{\varepsilon _k}(y) \vert \\&\le c\Vert \tilde{u}_{\varepsilon _k} \Vert _{C^0(\mathbb {R}^2)} \\&\le c \Vert \nabla \tilde{u}_{\varepsilon _k} \Vert _{L^{2,1}(\mathbb {R}^2)}, \end{aligned}$$

where we have used the below result.

### Lemma 3.1

(Theorem 3.3.4 [5]) If $u\in W^{1,2}(\mathbb {R}^2)$ with compact support and $\nabla u\in L^{2,1}(\mathbb {R}^2)$, then $u\in C^0(\mathbb {R}^2)$ with

$$\begin{aligned} ||u||_{C^0(\mathbb {R}^2)}\le c||\nabla u||_{L^{2,1}(\mathbb {R}^2)}. \end{aligned}$$

So we now only need to show $||\nabla \tilde{u}_{ \varepsilon _k}||_{L^{2,1}{(\mathbb {R}^2)}}\rightarrow 0$ to get the property (33).

This follows almost immediately from the proof of the energy bound. It remains to bound the normal part of $\nabla \tilde{u}_{ \varepsilon _k}$. Define $(\nabla \tilde{u}_{ \varepsilon _k})^\top$ and $(\nabla \tilde{u}_{ \varepsilon _k})^\bot$ to be the projections of $\nabla \tilde{u}_{ \varepsilon _k}(x)$ onto $T_{u(x)}N$ and $\mathcal {N}_{u(x)}N$ respectively. Then using Lemma 2.1 to take a spanning collection $\eta _i$ and the bound (30) we get

34 $$\begin{aligned} ||(\nabla \tilde{u}_{\varepsilon _k})^\top ||_{L^{2,1}(\mathbb {R}^2)}\le&c\sum _i||\langle \nabla \tilde{u}_{\varepsilon _k}, \eta _i u_{\varepsilon _k} \rangle ||_{L^{2,1}(\mathbb {R}^2)}\\ \le&c||\nabla u_{\varepsilon _k}||_{L^2(B_{\frac{R_0}{4}} \setminus B_{2Rt_k }))}+c\sqrt{\delta } \nonumber \end{aligned}$$

for *k* sufficiently large. Using (12) we see that

$$\begin{aligned} (\nabla \tilde{u}_{\varepsilon _k})^\bot =\big (\nabla (\varphi _{\frac{R_0}{16}}(x))\big )^\bot ( u_{\varepsilon _k}(x) - \bar{u}_{\varepsilon _k}^1) -\big (\nabla (\varphi _{4Rt_k}(x))\big )^\bot ( u_{\varepsilon _k}(x) - \bar{u}_{\varepsilon _k}^2) \end{aligned}$$

which we bound, identically to (28), using (13), (14) and (16)

35 $$\begin{aligned} ||(\nabla \tilde{u}_{\varepsilon _k})^\bot ||_{L^{2,1}(\mathbb {R}^2)}\le c\sqrt{\delta } \end{aligned}$$

for *k* sufficiently large. So combining the tangential, (34), and normal, (35), estimates gives

$$\begin{aligned} ||\nabla \tilde{u}_{\varepsilon _k}||_{L^{2,1}(\mathbb {R}^2)}\le c||\nabla u_{\varepsilon _k}||_{L^2(B_{\frac{R_0}{4}} \setminus B_{2Rt_k }))}+c\sqrt{\delta } \end{aligned}$$

for *k* sufficiently large. Then by using the energy identity, (7), and that $\delta >0$ was arbitrary, the no-neck property follows.

## A counterexample to the energy identity for $\varepsilon$-harmonic map sequences

We will now sketch an argument which will extend the counterexample to the energy identity for $\alpha$-harmonic maps, as constructed by Li and Wang in [15], to $\varepsilon$-harmonic maps. We can use the whole construction in their Section 3 up to their Lemma 3.5. In particular, we use the following. We may construct *N* as two copies of flat $\mathbb {T}^3$ attached together with a special metric defined by $\psi$ chosen in the neck region. Then a family of maps $u_k\in W^{1,2}(S^2,N)\cap C^0(S^2,N)$ may be constructed, with the properties that they are all pairwise non-homotopic and $\inf \limits _{u\in [u_k]}E_0[u]=8\pi \psi (0)$, where $[u_k]$ is the homotopy class of $u_k$. We also have the result that if *u* is a non-trivial harmonic map, with $E_0(u)<12\pi \psi (0)$ then $E_0(u)=4\pi \psi (0)$ or $8\pi \psi (0)$.

Now define $u_{\varepsilon ,k}\in [u_k]\cap W^{2,2}(S^2,N)$, for any $k\in \mathbb {N},\varepsilon >0$, to be a map which minimises the $\varepsilon$-energy within the homotopy class. Explicitly it satisfies

$$\begin{aligned} \tilde{E}_\varepsilon [u_{\varepsilon ,k}]=\inf \limits _{u \in [u_k]\cap W^{2,2}(S^2,N)} \tilde{E}_\varepsilon [u]. \end{aligned}$$

Existence follows from $\tilde{E}_\varepsilon$ obeying the Palais-Smale compactness condition.

### Lemma 4.1

For any $\lambda _0> 8\pi \psi (0)$, there exist sequences $\varepsilon _k \rightarrow 0$ and $i_k\rightarrow \infty$ such that

$$\begin{aligned} \tilde{E}_{\varepsilon _k} [u_{\varepsilon _{k,i_k}}] = \lambda _0 \quad \forall \; k \in \mathbb {N}. \end{aligned}$$

### Proof

First define for $\varepsilon \ge 0$

$$\begin{aligned} \varphi _k(\varepsilon ) = \inf \limits _{u \in [u_k]\cap W^{2,2}(S^2,N)} \tilde{E}_\varepsilon [u] \end{aligned}$$

and fix $\varepsilon _0>0$. Then we prove that for any fixed $\varepsilon \in (0,\varepsilon _0)$ the following holds

36 $$\begin{aligned} \lim \limits _{k\rightarrow \infty } \varphi _k(\varepsilon ) = + \infty . \end{aligned}$$

Suppose not, then we can find some subsequence $u_{\varepsilon ,k}$ with bounded $\varepsilon$-energy. By using Stokes’ theorem we note that that $L^2$-norm of $D^2u_k$ is uniformly bounded, and so indeed $||u_{\varepsilon ,k}||_{W^{2,2}}$ is uniformly bounded. Then by standard Sobolev embeddings and Morrey’s inequality we get $||u_{\varepsilon ,k}||_{C^{0,\alpha }}$ uniformly bounded for any $0<\alpha <1$ of our choosing. So then by Arzelà-Ascoli we may extract a subsequence $u_k\rightarrow u$ which converges in $C^0(S^2,N)$, but then $u_{\varepsilon ,k}\sim u$ for all *k* large enough. But by construction all $u_{\varepsilon ,k}$ are pairwise non-homotopic, giving a contradiction.

Further, we claim that for any fixed *k* we have $\varphi _k$ continuous on $[0,\varepsilon _0)$. Setting $0<\varepsilon _1< \varepsilon _2 < \varepsilon _0$ we first note that

$$\begin{aligned} \varphi _k(\varepsilon _1)\le \tilde{E}_{\varepsilon _1}[u_{\varepsilon _2,k}] \le \tilde{E}_{\varepsilon _2}[u_{\varepsilon _2,k}] = \varphi _k(\varepsilon _2) \end{aligned}$$

so $\varphi _k$ decreases as $\varepsilon \rightarrow 0$ and so is uniformly bounded for $\varepsilon \le \varepsilon _0$. We then have

$$\begin{aligned} \varphi _k(\varepsilon _1)=&\tilde{E}_{\varepsilon _1}[u_{\varepsilon _1,k}] \nonumber \\ =&\tilde{E}_{\varepsilon _2}[u_{\varepsilon _1,k}]-\frac{\varepsilon _2-\varepsilon _1}{2}\int \limits _{S^2} \vert \Delta u_{\varepsilon _1,k} \vert ^2 \nonumber \\ \ge&\varphi _k(\varepsilon _2)-\Big (\frac{\varepsilon _2-\varepsilon _1}{\varepsilon _1}\Big )\Big (\frac{\varepsilon _1}{2}\int \limits _{S^2} \vert \Delta u_{\varepsilon _1,k} \vert ^2\Big ) \end{aligned}$$

giving

$$\begin{aligned} 0\le \varphi _k(\varepsilon _2)-\varphi _k(\varepsilon _1)\le c\frac{\varepsilon _2-\varepsilon _1}{\varepsilon _1} \end{aligned}$$

which gives continuity on $(0,\varepsilon _0)$. Further, we show that $\varphi _k(\varepsilon )$ is right continuous at 0, i.e. $\lim \limits _{\varepsilon \searrow 0} \varphi _k(\varepsilon ) = \varphi _k(0)$. Fix $u \in W^{2,2}(S^2,N)$ and $\varepsilon >0$,

$$\begin{aligned} \varphi _k(0)\le E_0(u)\le \tilde{E}_\varepsilon (u) \end{aligned}$$

and taking the infimum over $u\in [u_k]\cap W^{2,2}(S^2,N)$ then $\varepsilon \rightarrow 0$ gives

$$\begin{aligned} \varphi _k(0)\le \lim \limits _{\varepsilon \searrow 0} \varphi _k(\varepsilon ). \end{aligned}$$

For the other inequality, note $u_k$ is smooth so, for any $\delta >0$, there exists a smooth map $u_k' \in C^\infty (S^2,N)$ in the same homotopy class such that

$$\begin{aligned} E_0[u_k'] \le \varphi _k(0) + \delta . \end{aligned}$$

Together with $E_0(u_k') = \lim \limits _{\varepsilon \searrow 0} \tilde{E}_\varepsilon [u_k']$ and the fact that $\varphi _k(\varepsilon ) \le \tilde{E}_\varepsilon [u_k']$ for each $\varepsilon$ we get

$$\begin{aligned} \lim \limits _{\varepsilon \searrow 0} \varphi _k(\varepsilon ) \le \varphi _k(0) + \delta \end{aligned}$$

so $\varphi$ is right continuous at 0. Equation (36) implies that given a sequence $\varepsilon _k\rightarrow 0$, we can take a sequence $i_k\rightarrow \infty$ such that $\varphi _{i_k}(\varepsilon _k)> \lambda _0$ for each *k*. Then by continuity of $\varphi$ and the fact that $\varphi _k(0)=8\pi \psi (0)$ for each *k* we can find some $0<\varepsilon _k'\le \varepsilon _k$ such that

$$\begin{aligned} \varphi _{i_k}(\varepsilon _k') = \tilde{E}_{\varepsilon _k'}[u_{\varepsilon _k', i_k}] = \lambda _0 \end{aligned}$$

completing the proof. $\square$

Analogously to Li and Wang, we can now construct a counterexample. Take a sequence constructed as in the lemma for some fixed $8\pi \psi (0)<\lambda _0<12\pi \psi (0)$. Then as the maps $u_{\varepsilon _k,i_k}$ are pairwise non homotopic, they must blow up as $k\rightarrow \infty$. Set $v_0$ to be the weak limit and $v_1,...,v_l$ the bubbles, these are all smooth homotopic maps with energy less than $12\pi \psi (0)$ so

$$\begin{aligned} \frac{1}{4\pi \psi (0)}(E_0[v_0]+\sum \limits _i^lE_0[v_i]) \end{aligned}$$

is an integer. However $\displaystyle \frac{\lambda _0}{4\pi \psi (0)}$ is not, therefore

$$\begin{aligned} \lambda _0=\lim _{k\rightarrow 0}\tilde{E}_{\varepsilon _k}[u_{\varepsilon _k,i_k}]\ne E_0[v_0]+\sum _i^lE_0[v_i] \end{aligned}$$

so the energy identity does not hold.

## Energy identity and no-neck property for a sequence of Sacks-Uhlenbeck maps to homogeneous spaces

We will now outline the argument for the $\alpha$-harmonic maps. This is done by generalising Li and Zhu’s [13] proof of Theorem 1.7 to the case where the target space is a homogeneous space. The only difference is the exact form of the conservation law used. As discussed earlier, we want to stress that since the $\alpha$-energy is an intrinsic property, the truth of this result will not depend on the existence of an equivariant embedding. However, this also means that the properties of a $\alpha$-harmonic map do not depend on the embedding, so we can freely assume that our target manifold is equivariantly embedded and it will not affect the properties.

The idea now is to give a proof of a variant of Noether’s Theorem formulated for $\alpha$-harmonic maps using the same $\eta$ as defined in Theorem 1.2. As in their paper, we assume that we are working on the flat unit ball *B*.

### Theorem 5.1

(Conservation law for $\alpha$-harmonic maps) Let $u_\alpha \in C^\infty (B,N)$ be an $\alpha$-harmonic map with *N* a homogeneous Riemannian manifold equivariantly embedded in $\mathbb {R}^l$ with $\Pi :G\rightarrow O(l)$ the associated Lie group embedding. Let $\eta$ be of the form $\eta = \frac{\partial }{\partial t}\Pi (\gamma (t)) \big |_{t=0}$ for some $\gamma (t)$ a smooth path in *G* with $\gamma (0)$ the identity. Then the following conservation law holds

37 $$\begin{aligned} {{\,\textrm{div}\,}}\;(F_\alpha \langle du_\alpha , \eta u_\alpha \rangle )&= 0 \end{aligned}$$

where $$F_\alpha = (1+\vert \nabla u_\alpha \vert ^2)^{\alpha -1}$$.

### Proof

Using the facts that $u_\alpha$ is an $\alpha$-harmonic map, so it satisfies (3), and that $\eta u_\alpha \in T_{u_\alpha }N$ we get

$$\begin{aligned} 0=&\langle {{\,\textrm{div}\,}}( F_\alpha d u_\alpha ) , \eta u_\alpha \rangle \nonumber \\ =&{{\,\textrm{div}\,}}\langle F_\alpha d u_\alpha , \eta u_\alpha \rangle - F_\alpha \langle d u_\alpha , \eta du_\alpha \rangle \nonumber \\ =&{{\,\textrm{div}\,}}(F_\alpha \langle d u_\alpha , \eta u_\alpha \rangle ) \end{aligned}$$

recalling that $\eta$ is a fixed anti-symmetric matrix. $\square$

Note that in the case of a non equivariant embedding we could have used Hélein’s $\rho$ instead here and this conservation law would still hold, with the proof of energy identity and the no-neck property following identically.

In the case of a round sphere, equipped with the traditional embedding, the isometry group is the whole orthogonal group, so $\eta$ can be any anti-symmetric matrix. In particular, by taking $$\eta _{a,b}={\left\{ \begin{array}{ll} 1, &  (a,b)=(i,j)\\ -1, &  (a,b)=(j,i)\\ 0, &  \text {otherwise}\\ \end{array}\right. }$$ we recover the family of conservation laws

$$\begin{aligned} {{\,\textrm{div}\,}}\big (F_\alpha (u_\alpha ^jdu_\alpha ^i-u^i_\alpha du^j_\alpha )\big )=0 \end{aligned}$$

obtained by Li and Zhu.

As discussed in Lemma 2.1 we can take a finite collection of $(\eta _{i}q)_{i=1}^I$ spanning $T_qN$ for all $q\in N$. Then by constructing locally and patching over by a partition of unity, or as in Lemma 2 of [4], one can construct *I* smooth tangent vector fields $(Y_i)_{i=1}^I$ on *N* such that for any $V\in \Gamma (TN)$ we have

$$\begin{aligned} V(q) = \langle V(q),\eta _{1}q\rangle Y_1(q)+...+ \langle V(q),\eta _{I}q\rangle Y_I(q). \end{aligned}$$

So in total we achieve a div-curl relationship,

$$\begin{aligned} {{\,\textrm{div}\,}}(F_\alpha \nabla u_\alpha )&= {{\,\textrm{div}\,}}(F_\alpha \sum \limits _{i=1}^I \langle \nabla u_\alpha , \eta _{i}u_\alpha \rangle Y_i(u_\alpha )) \nonumber \\&= \sum \limits _{i=1}^I {{\,\textrm{div}\,}}(F_{\alpha } \langle \nabla u_\alpha , \eta _{i}u_\alpha \rangle ) Y_i(u_\alpha ) + F_{\alpha } \langle \nabla u_\alpha , \eta _{i}u_\alpha \rangle \nabla (Y_i(u_\alpha )) \nonumber \\&= \sum \limits _{i=1}^I \langle \nabla ^\perp G_{\alpha ,i} , \nabla (Y_i(u_\alpha ))\rangle , \end{aligned}$$

where $G_{\alpha ,i}$ is defined to be a solution in $W^{1,2}(B)$ to $\nabla ^\perp G_{\alpha ,i} = F_{\alpha } \langle \nabla u_\alpha , \eta _{i}u_\alpha \rangle$. This further allows us to write, for $\tilde{u}_\alpha$ constructed as in step 2 of section 3 in [13],

$$\begin{aligned} {{\,\textrm{div}\,}}(F_\alpha \nabla \tilde{u}_\alpha )=&\sum \limits _{i=1}^I \langle \nabla ^\perp G_{\alpha ,i} , \nabla (Y_i(u_\alpha )\varphi _\delta (1-\varphi _{r_\alpha R})\rangle \\&+ {{\,\textrm{div}\,}}\Big (F_\alpha \big ( (u_\alpha -\bar{u}_\alpha ^1)\nabla \varphi _\delta -( u_\alpha -\bar{u}_\alpha ^2)\nabla \varphi _{r_\alpha R}\big )\Big ) \end{aligned}$$

which is analogous to their equation (3.13).

Using these results it is easy to obtain the result of no energy loss and no neck property by following the rest of the proof of Li and Zhu. The only terms that differ are the exact form of $G_{\alpha ,i}$ and in some formula we will have $Y_i(u_\alpha )$ instead of $u_\alpha ^i$, however it is easy to see that the required bounds will still follow. Indeed, all the required bounds on $G_{\alpha ,i}$ follow from the fact that $|\nabla G_{\alpha ,i}|\le c|F_\alpha |\cdot |\nabla u_\alpha |$ pointwise, which still holds in our case.

## Data Availability

No datasets were generated or analysed while completing this project.
